# Non-invasive vagus nerve stimulation attenuates proinflammatory cytokines and augments antioxidant levels in the brainstem and forebrain regions of Dahl salt sensitive rats

**DOI:** 10.1038/s41598-020-74257-9

**Published:** 2020-10-16

**Authors:** Madhan Subramanian, Laura Edwards, Avery Melton, Lyndee Branen, Angela Herron, Mahesh Kumar Sivasubramanian, Raisa Monteiro, Samantha Stansbury, Priya Balasubramanian, Lynsie Morris, Khaled Elkholey, Monika Niewiadomska, Stavros Stavrakis

**Affiliations:** 1 Department of Physiological Sciences, College of Veterinary Medicine, Oklahoma State University, 277 McElroy Hall, Stillwater, OK 74078 USA; 2 Reynolds Oklahoma Center On Aging, University of Oklahoma Health Sciences Center, Oklahoma City, OK USA; 3 Department of Medicine, Cardiovascular Section, University of Oklahoma Health Sciences Center, Oklahoma City, OK USA; 4 Department of Medicine, Heart Rhythm Institute, University of Oklahoma Health Sciences Center, 800 Stanton L Young Blvd, Suite 5400, Oklahoma City, OK 73104 USA

**Keywords:** Cardiovascular biology, Neurophysiology, Molecular neuroscience

## Abstract

The anti-inflammatory effects of vagus nerve stimulation are well known. It has recently been shown that low-level, transcutaneous stimulation of vagus nerve at the tragus (LLTS) reduces cardiac inflammation in a rat model of heart failure with preserved ejection fraction (HFpEF). The mechanisms by which LLTS affect the central neural circuits within the brain regions that are important for the regulation of cardiac vagal tone are not clear. Female Dahl salt-sensitive rats were initially fed with either low salt (LS) or high salt (HS) diet for a period of 6 weeks, followed by sham or active stimulation (LLTS) for 30 min daily for 4 weeks. To study the central effects of LLTS, four brainstem (SP5, NAb, NTS, and RVLM) and two forebrain sites (PVN and SFO) were examined. HS diet significantly increased the gene expression of proinflammatory cytokines in the SP5 and SFO. LLTS reversed HS diet-induced changes at both these sites. Furthermore, LLTS augmented the levels of antioxidant Nrf2 in the SP5 and SFO. Taken together, these findings suggest that LLTS has central anti-inflammatory and antioxidant properties that could mediate the neuromodulation of cardiac vagal tone in the rat model of HFpEF.

## Introduction

Heart failure with preserved ejection fraction (HFpEF) is one of the leading causes of mortality in the elderly^1^. The prevalence of HFpEF increases from 1% at age 40 to about 10% at age 80^2^. It is the most common cause of hospitalization in patients ≥ 65 years of age and with the growing elderly population, these trends are expected to worsen^2,3^. The prognosis for patients with HFpEF is poor (5-year mortality rate approximately 50%)^4^, and so far, no treatment has been demonstrated to achieve positive outcomes on morbidity or mortality in these patients^5^. Inflammation and fibrosis are the most prominent pathophysiological mechanisms that mediate impairments of cardiac function in heart failure. Identification of treatment options that target these cardiac mechanisms may prove fruitful.

Vagus nerve stimulation (VNS) is a non-pharmacological, device-based therapy that is currently approved by FDA for the treatment of epilepsy and depression^6^. Recent studies have indicated that low-level VNS suppresses atrial fibrillation and inflammatory cytokines in humans^7–9^. In addition, several preclinical studies have yielded promising results for the use of non-invasive VNS in improving cardiac outcomes of HFpEF^10,11^. Using Dahl salt sensitive rats on high salt diet as a rodent model for HFpEF, it was recently shown that chronic, intermittent, low-level, tragus stimulation (LLTS) significantly ameliorated diastolic function and attenuated left ventricular (LV) inflammation and fibrosis^10^. Although it is well-established that the auricular branch of the vagus nerve (ABVN) is activated during LLTS, the neural pathways and central mechanisms underlying the effects of LLTS on inflammation and heart function have not been fully explored^12^. In fact, previous studies have shown that increased oxidative stress and inflammation in the brains of Dahl sensitive rats contribute to autonomic imbalance. This has been characterized by increased sympathetic nerve activity^13^, which is known to play a critical role in the progression of heart failure. However, there is a significant gap in knowledge in understanding the neural circuitry that is modulated by LLTS, which in turn may lead to identification of potential therapeutic targets.

The sensory afferents from ABVN innervate two brainstem regions, the nucleus tractus solitarius (NTS) and spinal trigeminal nucleus (SP5)^14–17^ and hence present the possibility for the involvement of both these neural centers in LLTS mediated beneficial effects on the heart. The first possibility may involve activation of the NTS by ABVN afferents to inhibit the rostral ventrolateral medulla (RVLM) via stimulation of the caudal ventrolateral medulla (CVLM), resulting in reduced sympathetic output to the heart. This is a well-known arterial baroreflex pathway that provides arterial pressure stabilization through feedback control of cardiac and vascular tone^18,19^. The second possibility might involve activation of either NTS or SP5 by ABVN afferents resulting in stimulation of vagal efferents (cardiac vagal preganglionic parasympathetic neurons) from the nucleus ambiguus (NAb) to inhibit heart rate^15,20^. In addition to the brainstem, studies involving direct vagus nerve stimulation have shown c-FOS immunoreactivity in forebrain regions such as the paraventricular nucleus (PVN) and subfornical organ (SFO), to which direct neuronal projections from NTS has been well established^21,22^. Whether LLTS acts through any of these neural circuits to exert beneficial effects on the heart is not known.

The overall objective of this study was to understand the effects of LLTS on four brainstem (SP5, NAb, NTS and RVLM) and two forebrain regions (PVN and SFO) that are important for the regulation of cardiac vagal tone^17^ by using a well-established rat model of HFpEF (Dahl salt sensitive rats)^23^. The hypothesis behind this study is that LLTS would attenuate the pro-oxidant and pro-inflammatory response exerted by HS diet in the brainstem regions that are part of the ABVN-heart neural circuitry.

## Methods

### Experimental animals and treatment

Fourteen female DS rats were purchased from Charles River Laboratories (Wilmington, MA, USA) and were maintained on 0.3% NaCl (low salt, LS) diet until 7 weeks of age. After 7 weeks, the animals were randomly divided into three groups. All the animals in the first group (n = 4) continued to be fed with LS diet until the end of the experimental period. The remaining animals (n = 10) were fed with 8% NaCl (high salt, HS) diet for 6 weeks. After 6 weeks of HS, animals received either sham (n = 6) or active (n = 4) LLTS treatment for an additional 4 weeks. Heart rate, blood pressure (BP) and body weight were determined at 6 weeks after LS/HS diet (baseline) and 4 weeks after LLTS treatment (endpoint). BP measurements were obtained non-invasively using the tail-cuff method (AD Instrument, PowerLab Data Acquisition System, New South Wales, Australia). Echocardiography (Acuson SC2000, Siemens) was performed at the same time points to assess diastolic function, under isoflurane 2% anesthesia as described previously^10^. At the end of 4 weeks of LLTS sham or active treatment, the animals were euthanized with 5% isoflurane by inhalation and the brainstems were harvested for analysis. The final LLTS was performed about 24 h before the euthanasia. Water was provided ad libitum throughout the duration of the treatment. All the animal protocols were approved by the Institutional Animal Care and Use Committee of the University of Oklahoma Health Science Center (protocol number 16‐106) and were carried out under the guidelines of the National Institutes of Health Guide for the Care and Use of Laboratory Animals.

### LLTS

LLTS (20 Hz frequency, 0.2 ms pulse duration, 2 mA amplitude) was given through a TENS device (InTENSity Twin Stim, Current Solutions LLC, Austin, TX, USA) daily for 30 min over a 4-week period under 2% isoflurane anesthesia as described previously^10^. Briefly, in HS sham group, the electrodes were placed on the auricular margin which does not have any vagal innervation whereas in HS active group they were placed over the auricular concha region to stimulate the auricular branch of vagal nerve.

### Heart rate variability

At baseline, a 3-min ECG was recorded before and during active stimulation in all the rats. Heart rate variability (HRV) in the time domain (standard deviation of NN interval, SDNN and root mean square of consecutive NN intervals, RMSSD) and in the frequency domain (low frequency, LF, 0.2–0.8 Hz and high frequency, HF, 0.8–2.5 Hz), was calculated, as previously described^24^, using the Kubios software.

### Microdissection of key brainstem nuclei and forebrain regions

Serial coronal sections (300 μm thick) of the brainstem were obtained on super frost plus slides using a cryostat. The spinal trigeminal nucleus (SP5), nucleus ambiguus (NAb), nucleus tractus solitarius (NTS), rostral ventrolateral medulla (RVLM), paraventricular nucleus (PVN) and subfornical organ (SFO) were microdissected by Palkovits’s technique. A rat brain stereotaxic atlas was used as a reference (Paxinos and Watson, 1987).

### Real time PCR

Micropunches from brain regions were collected from either side of the 300 μm thick section based on the stereotaxic co-ordinates. Punches were collected from the SP5, NAb, NTS, RVLM, PVN, and SFO regions and used for RNA extraction and real-time PCR analysis. The tissue micropunches were lysed in Trizol reagent and RNA was extracted using Direct-zol RNA Microprep kit (ZymoResearch, Irvine, CA, USA). cDNA was synthesized using High-Capacity cDNA Reverse Transcription Kit (Applied Biosystems). Real time PCR reactions were carried out using iTaq Universal SYBR Green mix (Biorad) with 10 ng cDNA per reaction. The rat primers for IL6, IL1-Beta, TNF-Alpha, MCP1, CXCL1, Nrf2, NQO1, Fra1 and GAPDH (the housekeeping gene) were designed using primer blast software and synthesized by Integrated DNA technologies (primer sequences are listed in Table 1). Data was analyzed by 2^−ΔΔCT^ method.

**Table 1:** Primer sequences and their gene accession numbers for real time PCR analysis are listed.

Rat gene	Forward primer	Reverse primer	Gene accession number
IL6	CTGGTCTTCTGGAGTTCCGT	TGCTCTGAATGACTCTGGCT	NM_012589.2
IL-1Beta	GACTCGTGGGATGATGACGAC	GAGCTTTCAGCTCACATGGGT	NM_031512.2
TNF-Alpha	ACCATGAGCACGGAAAGCAT	CTTGTTGGGACCGATCACCC	NM_012675.3
MCP1	CACTCACCTGCTGCTACTCATT	TACAGCTTCTTTGGGACACCTG	NM_031530.1
CXCL1	GCGCCCGTCGCCAAT	GGCATCACCTTCAAACTCTGGA	NM_030845.1
Nrf2	AGAGCAACTCCAGAAGGAACAG	TTGGGAGGAATTCTCCGGTCT	NM_031789.2
NQO1	ATTGTATTGGCCCACGCAGA	GATTCGACCACCTCCCATCC	NM_017000.3
Fra-1	TGGATGGTGCAGCCTCATTT	GCTCGTATGACTCCTGGTCG	NM_012953.1
GAPDH	GGGTGCCAACCCCAAACGTA	CCATAGGTCCCTTGGCTGCT	NM_023964.1

### Statistical analysis

All statistical analysis was performed using GraphPad Prism Version 8.3. All data were analyzed using one-way ANOVA followed by post hoc Tukey’s multiple comparison test. Log transformation of the heart rate variability parameters was performed to achieve normality and a paired t-test was used for comparison. All gene expression values were expressed as fold change relative to low salt. A p-value of < 0.05 is considered statistically significant.

### Ethical approval

This article does not contain any study with human participants. The experimental protocols using rats were approved by the Institutional Animal Care and Use Committee of the University of Oklahoma Health Science Center (protocol number 16‐106) and were carried out under the guidelines of the National Institutes of Health Guide for the Care and Use of Laboratory Animals.

## Results

### Effects of LLTS on HFpEF phenotype

After 6 weeks of treatment with HS diet, rats developed hypertension, left ventricular hypertrophy and diastolic dysfunction [as indicated by elevated early mitral inflow velocity to early diastolic mitral annulus velocity (E/e’)], compared to LS rats (158.3 ± 12.6 mmHg vs. 123.8 ± 9.8 mmHg, p = 0.0004; 2.4 ± 0.2 mm vs. 1.8 ± 0.1 mm, p = 0.02; 24.0 ± 2.1 vs. 18.3 ± 1.6, p = 0.01, respectively). Consistent with our previous study, 4 weeks of LLTS attenuated the increase in BP (146.0 ± 10.2 mmHg vs. 183.5 ± 9.8 mmHg, respectively, p = 0.04), left ventricular thickness (2.3 ± 0.1 mm vs. 2.7 ± 0.1 mm, p = 0.03, respectively) and diastolic dysfunction (18.8 ± 3.0 vs. 26.5 ± 4.9, p = 0.01, respectively) induced by HS diet, without any change in left ventricular ejection fraction (p > 0.05).

### Effects of LLTS on cytokine expression in the brainstem and forebrain regions

**Figure 1:**
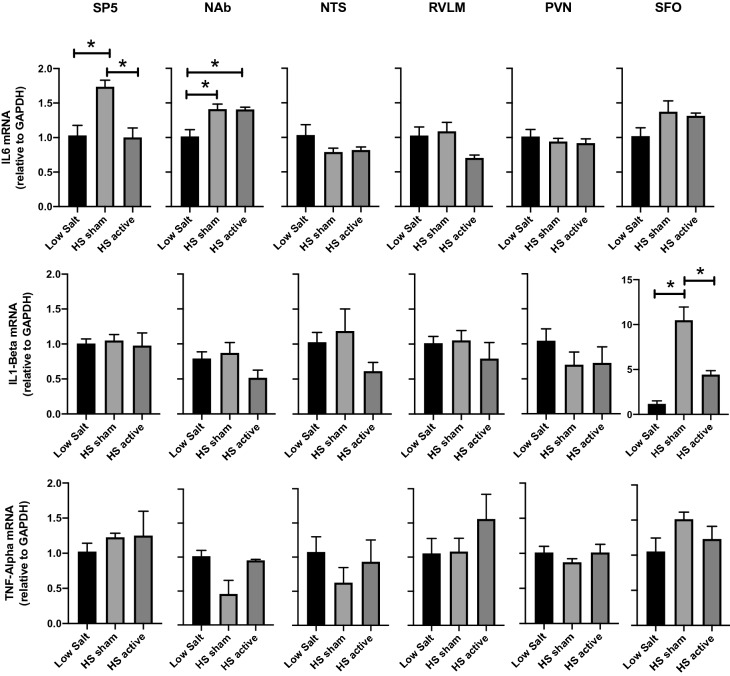
Gene expression of cytokines in the brainstem and forebrain regions after LLTS or sham stimulation. The mRNA levels of cytokines IL-6, IL1-Beta and TNF-Alpha in the brain regions SP5, NAb, NTS, RVLM, PVN, and SFO after low salt (LS), high salt with sham stimulation (HS sham) and high salt with LLTS (HS active) treatment are shown. Values are mean ± SE (n = 4–6/group). *P < 0.05 is considered statistically significant.

To determine the central effects of LLTS on inflammation, the gene expression of cytokines IL-6, IL1-Beta and TNF-Alpha was measured in four brainstem (SP5, NAb, NTS, and RVLM) and two forebrain (PVN and SFO) regions after HS treatment. High salt resulted in significant increases in the level of IL-6 (Fig. 1) in the SP5 and NAb compared to low salt (LS) controls. LLTS attenuated the increases in IL-6 expression in HS rats in the SP5 but not in the NAb region. There were no significant changes in IL1-Beta or TNF-Alpha levels in SP5 and NAb. There was a tenfold increase in the expression of IL-1Beta in the SFO of HS group compared to low salt. LLTS was able to significantly reduce the IL-1Beta in the SFO. No changes were observed in the expression of both of these inflammatory markers in the NTS, RVLM, and PVN among different groups.

### Effects of LLTS on chemokine expression in the brainstem and forebrain regions

**Figure 2:**
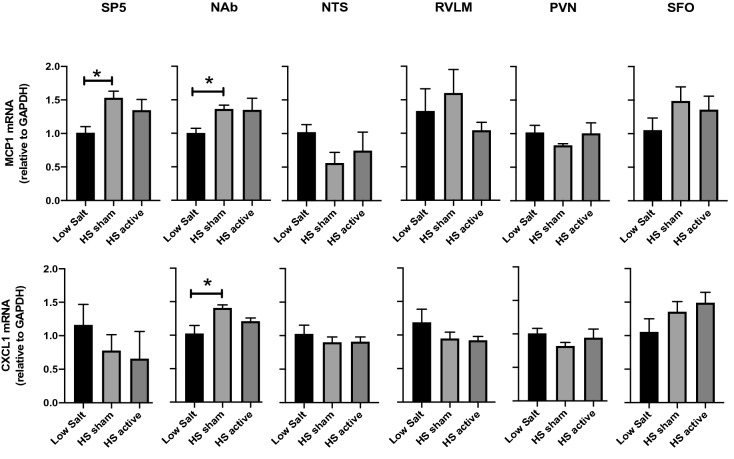
Gene expression of chemokines in the brainstem and forebrain regions after LLTS or sham stimulation. The mRNA levels of chemokines MCP1 and CXCL1 in the brainstem regions SP5, NAb, NTS and RVLM and forebrain regions PVN and SFO after LS, HS sham and HS active treatment are shown. Values are mean ± SE (n = 4–6/group). *P < 0.05 is considered statistically significant.

To elucidate the central effects of LLTS on chemokines, we measured the gene expression of MCP1 and CXCL1 after HS treatment. HS rats showed significant increase in the mRNA levels of MCP1 in both SP5 and NAb compared with LS controls (Fig. 2). However, LLTS failed to attenuate MCP1 expression in these brainstem regions. Similarly, CXCL1 was upregulated in the NAb of HS sham rats and LLTS was not able to reduce its expression. Neither HS diet nor LLTS had any effects on chemokine expression in the other regions that were tested.

### Effects of LLTS on antioxidant expression in the brainstem and forebrain regions

**Figure 3:**
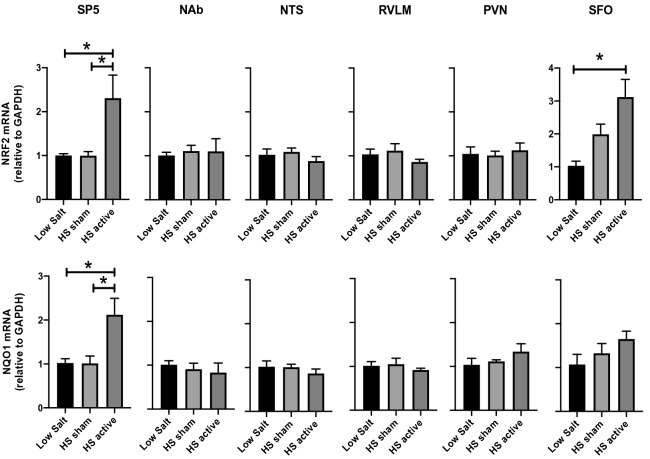
Gene expression of antioxidants in the brainstem and forebrain regions after LLTS or sham stimulation. The mRNA levels of antioxidants Nrf2 and NQO1 in the brain regions SP5, NAb, NTS, RVLM, PVN and SFO after LS, HS sham and HS active treatment are shown. Values are mean ± SE (n = 4–6/group). *P < 0.05 is considered statistically significant.

It is widely accepted that vagus nerve stimulation has systemic antioxidant properties^25,26^. To test whether LLTS has any central antioxidant effects, mRNA expression of the antioxidant gene, NQO1 and its transcriptional regulator Nrf2 were measured in four brainstem and two forebrain regions. HS diet did not produce any changes in the expression of Nrf2 or NQO1 in the SP5 compared to LS controls. However, LLTS produced a significant upregulation of Nrf2 and NQO1 in the SP5 compared to both LS and HS sham (Fig. 3). Similar increases in Nrf2 expression were also seen in the SFO region of the LLTS group suggesting LLTS may be involved in the activation of the antioxidant pathways. Interestingly, LLTS mediated upregulation of antioxidant gene expression was specific only to SP5 and SFO and not observed in the other regions that were tested (NAb, NTS, RVLM, and PVN).

### Effect of LLTS on markers of neuronal activity in the brainstem and forebrain regions

**Figure 4:**
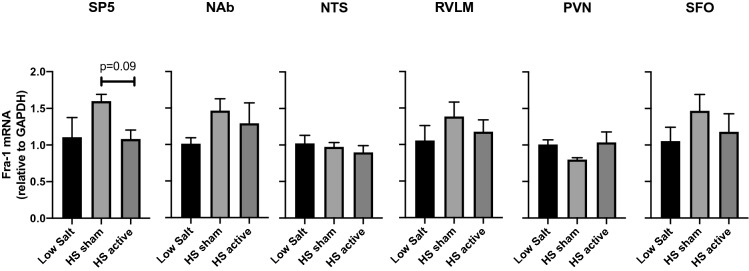
Gene expression of neuronal activity markers in the brain regions after LLTS or sham stimulation. The mRNA levels of markers Fra-1 in the brain regions SP5, NAb, NTS RVLM, PVN, and SFO after LS, HS sham and HS active treatment are shown. Values are mean ± SE (n = 4–6/group). *P < 0.05 is considered statistically significant.

The gene expression of molecular marker of long-term neuronal activity namely Fra-1 was measured in the regions of interest. A numerical trend was observed towards upregulation of Fra-1 gene in the HS sham group followed by an inclination towards downregulation in both the SP5 and SFO regions within the LLTS treated group. However, the data did not reach statistical significance (Fig. 4).

### Acute effects of LLTS on autonomic tone

**Figure 5:**
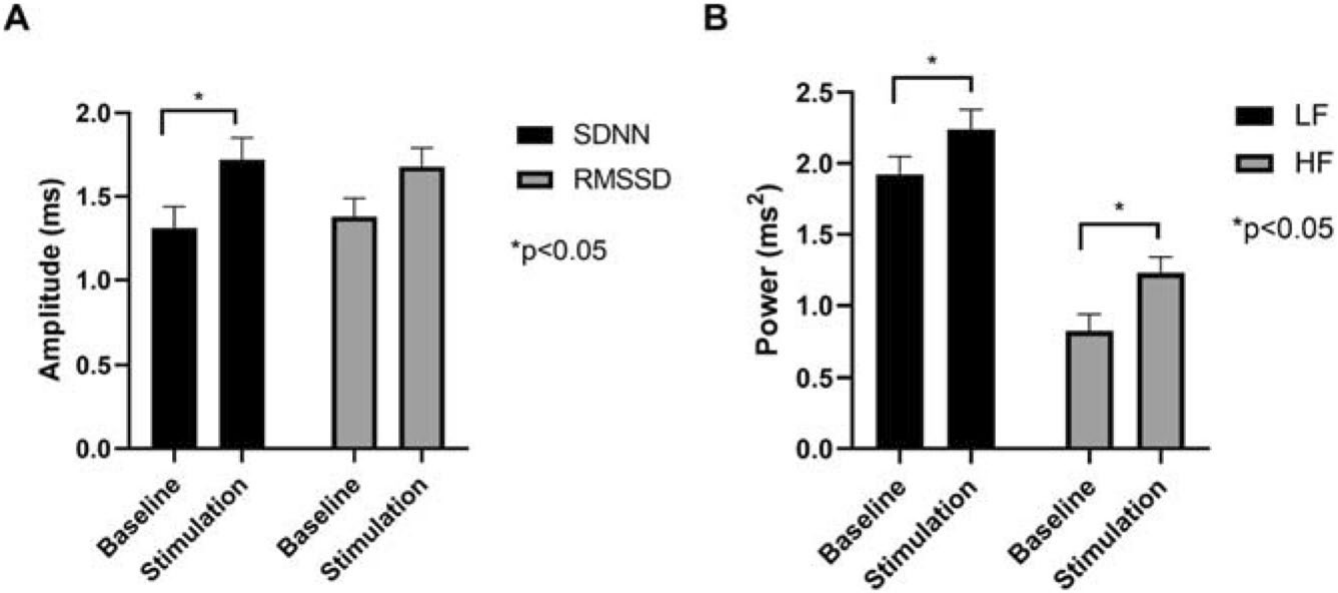
Acute effects of LLTS on heart rate variability parameters. The amplitude of time domain parameters (SDNN and RMSSD) and the power of frequency domain parameters (LH and HF) are shown. Values are mean ± SE. *P < 0.05 is considered statistically significant.

Heart rate variability was used to assess the acute effects of LLTS on autonomic tone, in order to ensure that LLTS resulted in stimulation of parasympathetic elements. Both SDNN and RMSSD were measured before (baseline) and during active LLTS (stimulation) indicating the long and short-term variations in heart rate respectively. Compared to baseline, SDNN was significantly upregulated during LLTS stimulation. RMSSD showed an increase during active stimulation, however it did not reach statistical significance (Table 2). Both LF and HF powers were increased during LLTS, consistent with acute cardiac autonomic modulation (Fig. 5). Acute changes in heart rate variability may not reflect long-term effects of LLTS. However, it has recently been shown that chronic LLTS results in changes in HRV in humans^9^.

**Table 2:** Heart rate variability parameters before and during stimulation are shown.

Variable	Baseline	Stimulation	P-value
SDNN (log) (ms)	1.31 ± 0.13	1.72 ± 0.13	0.04
RMSSD (log) (ms)	1.38 ± 0.11	1.68 ± 0.11	0.09
Low frequency (log) (ms^2^)	1.92 ± 0.13	2.24 ± 0.14	0.04
High frequency (log) (ms^2^)	0.83 ± 0.11	1.23 ± 0.11	0.01

## Discussion

The effects of LLTS on the central neural circuits involved in the autonomic control of heart are not well established. In order to investigate the neural circuitry mediating the effects of LLTS on the heart, four brainstem and two forebrain regions were analyzed for alterations in the gene expression of pro-inflammatory and antioxidant markers after 4 weeks of LLTS in a rat model of HFpEF. Results showed that HS diet exerted pro-inflammatory effects marked by increased levels of IL6, MCP1, and CXCL1 specifically in the SP5 and NAb of the brainstem and IL-1Beta in the SFO of the forebrain. LLTS attenuated inflammation by decreasing IL6 levels in the SP5 and IL-1Beta in the SFO. In addition, LLTS stimulated antioxidant defense mechanisms by activating the Nrf2 pathway in the SP5 and SFO of HS diet animals. These results are consistent with a recent study which showed that the cardiovascular autonomic effect of tragus stimulation is mediated at least partly through spinal cervical sensory afferent pathways, including SP5^16^. Moreover, only sparse labeling of transganglionic tracer injected into the tragus of rats was found in NTS^16^. No strong evidence was found to support the direct involvement of NTS in LLTS mediated autonomic regulation of the heart. The parallel observations in the gene expression of antioxidant, anti-inflammatory and neuronal activity markers seen in the SFO similar to SP5 is novel. The data suggests that ascending inputs from brainstem site (possibly NTS) may function in modulating the response of neurons within the circumventricular organ (SFO) which in turn could be responsible for the observed alterations in blood pressure and heart rate variability after LLTS^22,27^. These results provide preliminary evidence that LLTS modulates central neural circuits, specifically at the level of SP5 and SFO, and challenge the notion that LLTS activates only the auricular branch of the vagus nerve^17^. On the other hand, the LLTS effects on heart rate variability, which are consistent with acute cardiac autonomic modulation, support the notion that stimulation engages central vagal pathways. Further studies are warranted to delineate the exact anatomical basis of transcutaneous VNS, in order to better guide future clinical trials.

Numerous studies showed that LLTS had anti-inflammatory effects in a variety of cardiovascular diseases^7,28^. These effects were attributed to activation of the cholinergic anti-inflammatory pathway, which uses the vagus nerve as the efferent limb^29^. Recently it has been shown that LLTS alleviates cardiac inflammation and fibrosis in a rat-model of HFpEF, but the neural mechanisms behind the protective effects of LLTS remain unclear^10^. In the present study, HS diet significantly increased the proinflammatory cytokine IL-6 mRNA in the Dahl salt sensitive rats in both SP5 and Nab, but not in other regions tested. The result showed that HS diet produces inflammation in key brainstem regions (SP5 and NAb) important for cardiac vagal control. After LLTS in HS rats, IL-6 mRNA was significantly downregulated in the SP5 suggesting that LLTS reduces inflammation at the brainstem level. One possible mechanism by which LLTS reduces cardiac inflammation in this HFpEF model could be through modulation of autonomic activity in key brainstem regions involved in cardiac vagal tone. Notably, in the present study, HS diet induced upregulation of IL-6 was observed only at SP5 but not in NTS. LLTS was able to significantly attenuate the increase in IL-6 mRNA. Interestingly, a significant upregulation in IL-6 mRNA was also noted at NAb after HS diet, however LLTS was not able to reverse this increase. NAb is downstream from SP5 and NTS. Afferent signals from LLTS through ABVN first reaches SP5 and NTS, signals from there could be transmitted downstream by direct neuronal connection between these regions and NAb^14,20^. It has been well documented that NAb neurons directly innervate heart and are involved in the modulation of cardiac vagal tone^20^.

In addition to inflammatory cytokines, the effects of LLTS on central chemokines after HS treatment in rats was not known. In a recent human study, non-invasive transcutaneous VNS decreased both cytokines and chemokines from whole blood culture^30^. Furthermore, VNS has been shown to decrease chemokines in rat model of cardiac ischemia and after ischemia–reperfusion injury^31,32^. In the present study, HS treatment significantly upregulated the mRNA expression of chemokines MCP1 in SP5 and NAb and CXCL1 in the NAb region. In terms of chemokines, there was no statistically significant difference detected between the HS sham and HS active groups. Beneficial effects of LLTS in SP5 and NAb were only restricted to cytokine expression and not chemokines, which is intriguing. Significant changes were not observed in the chemokines tested at the level of NTS, RVLM, PVN and SFO. Taken together, these results demonstrated that LLTS attenuate HS-induced inflammation in a key brainstem and forebrain region, however further studies are needed to confirm these findings.

Central oxidative stress is characterized by accumulation of excess free radicals from reactive oxygen or reactive nitrogen species and has been implicated in several pathological conditions including HS-induced heart failure^33^. Nrf2 is a master transcription factor that regulates the expression of antioxidant and anti-inflammatory genes^34,35^. The present study explored whether LLTS activated the central antioxidant pathway in order to reduce the HS-induced oxidative stress. Even though oxidative stress was not measured in the present study, it has been well established by numerous other studies using similar animal model^33,36^. The present study found that LLTS significantly upregulated the mRNA of Nrf2 in the SP5 and SFO suggesting that LLTS increases antioxidant capacity at the central level. Interestingly, these increases in Nrf2 and NQO1 after LLTS were not observed in other brain regions examined.

## Clinical implications

The importance of this data is highlighted by the fact that therapies that improve outcomes in patients with HFpEF are lacking at present^5^. The study results support the notion that autonomic modulation may be used as a novel non-pharmacological therapeutic modality for HFpEF. Consistent with this notion, an ongoing clinical study (ANTHEM-HFpEF) aims at evaluating the feasibility, tolerability and safety of right cervical VNS in patients with symptomatic HFpEF^37^. In addition, understanding the effects of LLTS on key brainstem regions that are important for the regulation of cardiac vagal tone may lead to identification of novel targets for therapy in this patient population.

## Limitations

One limitation of the current study is that changes induced by LLTS in the brainstem and forebrain regions were not studied at the protein level. The relatively small amount of tissues dissected from each brain region in the present study is the reason for this limitation. In order to uncover which brain regions are actively involved during LLTS, the gene expression of long-term neuronal activity marker Fra-1 was measured. Even though numerical trends were seen, statistically significant differences were not observed between the groups. This is most likely due to decreased power of the study to detect changes in Fra-1 expression and therefore unable to positively confirm the exact brain regions involved while using this approach. Another caveat of the present study is that even though four brainstem and two forebrain regions were tested, other regions that are immunoreactive to vagal nerve stimulation are not examined^21^. The stimulation parameters used in the study were derived from previous experimental^10^ and human studies^38^. The effect of different stimulation parameters were not systemically assessed on outcomes, as such an assessment was beyond the scope of this study. In light of the disappointing results of VNS in patients with heart failure, despite the favorable preclinical data and the clear rationale for correcting the sympathovagal imbalance in this disease^39^, further research is necessary to optimize the stimulation parameters of autonomic modulation therapies, including VNS and LLTS. Finally, the current study is only able to show an association between the LLTS and its beneficial effects on the antioxidant and anti-inflammatory properties in the brainstem and forebrain. More mechanistic studies are warranted in the future to confirm the functional relevance of our current findings.

## Conclusion

In summary, the central pathways by which LLTS could attenuate HFpEF phenotype in Dahl salt sensitive rats was evaluated in this study. The protective mechanism of LLTS in this model might be associated to the reduction of inflammatory and enhancement of antioxidant capacity in the brainstem (SP5) and forebrain (SFO) regions thus modulating the cardiac vagal tone.
